# Record of Medical Science

**Published:** 1847-05

**Authors:** 


*Abstract of a Letter from* DR. N. S. JARVIS, Surgeon U. S. Army, dated Monterey, Mexico, Oct., 1846, embracing several Surgical Cases, which fell under his Treatment and Observation.

After stating matters of a private nature, Dr. Jarvis continues: "On the 19th of September we encamped within four miles of Monterey, in a grove of Peccan trees, called "Walnut Grove," where we were abundantly supplied with clear and cold water, from a stream of considerable size and rapidity, formed by the junction of numerous springs, which took their rise in the surrounding lime-stone rocks. The combination of wood and shade rendered this spot admirably fitted for an encampment. On the following day parties were employed in reconnoitring the enemy, and in observation of the fortified position of the town. Towards evening my Regiment, 3d Infantry, with another, were advanced a mile towards the town, to cover a party of engineers, engaged in the erection of a mortar battery, but returned to camp about 9, P. M., having been relieved by another regiment.

On the morning of the 21st the whole division was thrown forward towards the city, with a view, as we supposed at the time, of making a diversion in favor of the 2d division, under Gen. Worth, which was moving on the western side of the city by the Saltillo road. Few of us supposed, as we silently marched along, occasionally passing through corn fields and by the side of hedges, or whatever could conceal our movements from the enemy in their batteries, that we should so shortly be engaged in a fierce and deadly strife. As soon as, or in fact before, we emerged from under cover, the batteries from either end of the city opened their fire upon us, completely sweeping the plain in every direction, and enfilading the advancing columns of our troops, now rapidly marching towards the suburbs. The engineer officer having reported the practibility of attacking with success the rear of some of their forts, the 1st, 3d, and 4th Infantry were ordered to advance rapidly by separate roads, and now it was my professional labors commenced ; the nearest and only shelter that presented itself to me for the wounded, falling every moment under a most destructive fire, was a quarry pit, four or five feet in depth, and the same in breadth. Several of these were contiguous, and to them I directed the wounded to be carried. By stooping we were protected from the shots, which, however, became every moment thicker, owing to the fact, that our troops had by this time advanced within range of the enemy's fire, and the moment they perceived a party of men bringing the wounded to us, they directed all their guns upon it. I had already performed one amputation, and was preparing for a second, when two or three fugitives rushed into the pit, falling over the wounded that lay there crowded together, saying that a large body of lancers were approaching. So little credit did I attach to their report, which I ascribed rather to their fears than the actual presence of this dreadful description of troops, that I never raised my eyes to observe them ; which circumstance doubtless saved us all. Had I been discovered, all would have been massacred, as in their headlong fury, they would neither have delayed to ascertain our character or profession, nor have paid much respect to our patients. Several soldiers who had sought an adjoining pit with an officer were slain. They were soon after repulsed by a regiment of Ohio and Mississippi Volunteers, marching to reinforce those already in the town, and their retreat was farther quickened by a shower of grape opened upon them by our artillery.

I commenced with a determination of giving you a surgical history of the actions of the 21st, 22d, and 23d September, but have unintentionally thus far given a military narrative. This, however, will show, in the incidents above narrated, that the military surgeon is at times somewhat unpleasantly situated, when in the discharge of his professional duties, deprived as he is of the security, and many of the appliances enjoyed by his fellow practitioner in civil life.

The first wounds were received in crossing the plain, and were inflicted by grape and cannon-shot. This was of course before we had approached within reach of their musketry. These wounds were all low : generally at, or just above the ankle, according to distance and direction. Of the first three men brought to me, two had received wounds from twelve pound shots just above the ankle, which had nearly severed the limbs, which were hanging only by a portion of integuments. The other had his heel torn off by a six pound shot. Shortly after, our troops having advanced within reach, and under the fire of the Mexican Infantry, numerous cases of wounds by musket and escopette* balls were brought to me ; these latter are one-third

* An escopette is a short carbine, similar to a blunder-buss, and carries a ball one-third larger than our musket.—M.

larger than our musket-balls, and consequently inflict a more severe and formidable wound. So numerous at this time became the wounded in our pit, and so constant and heavy the fire, directed towards the parties approaching with the wounded, as to compel us to remove our hospital several hundred yards farther in the rear. We had not long been in our new position, when some covered wagons bringing the wounded attracted the attention of the enemy, who immediately re-opened their fire, compelling us a second time to remove beyond the range of their shot.

Among the numerous projectiles, occasioning severe and fatal wounds, were grape, canister, fragments of iron and copper shells, and stones knocked by the balls from the buildings and walls. Their shells were thrown with great accuracy, frequently in the midst of a body of troops, but fortunately killing and wounding but few.

Before speaking of any particular wounds, I will here take occasion to make some remarks respecting the character they assumed, and the peculiar causes acting to prevent a favorable result, so far as regarded the healing of all, even the most slight. The first annoyance we experienced, and which no doubt exerted an injurious effect, was one little anticipated at the time. The moment a limb was amputated numerous flies would alight on the stump, and must have deposited their eggs, for when it became necessary to dress the stump, myriads of maggots were found buried in it, which could be expelled with great difficulty ; rendering it necessary in some instances to re-open the flap, for their complete extermination. A much more formidable enemy made its appearance in an erysipelatous inflammation of the integuments, covering the stump, which generally set in two or three days after the operation ; and notwithstanding all the means made use of to arrest it, most commonly ended in sloughing, and either proved fatal or rendered a second amputation necessary. That some influence existed previously, either external or internal, from causes connected with the state of the atmosphere, or habits of the men, arising from diet or water, was manifest. The slightest wound or scratch became in every case a tedious ulcer, in some instances proving a cause for serious alarm. Apparently the most trifling wounds required an unusual time for healing, and even those that had previously healed would break out again, and present greater difficulty in their cure than in the first instance.

At this period no atmospheric causes apparently existed to produce this unfavorable aspect of things. Nothing could exceed the loveliness of the weather, if I may so express myself, and if the middle of the day were warm, the morning and evening refreshed us by a most delightful temperature and cloudless sky. No rain had fallen, with the exception of one or two showers, for nearly a month, and consequently little moisture existed to produce its well-known morbific influence. Immediately after the capitulation of the city, on the 25th of September, all the wounded of the different divisions entered the town, and suitable buildings were provided for their accommodation. Upwards of two hundred officers and men from the 1st and 3d

Divisions, who had been most severely wounded, were conveyed thither on the same day in litters and wagons. The wounded of the 2d Division already occupied the city.

Our camp afforded no comfort nor shelter for them beyond a few small tents and a solitary blanket laid on the ground: and many were destitute of even this apology for a bed, having lost them on our march. Many had no other clothing than that in wear, which was not only torn and soiled in climbing over the hedges, walls, &c., during the battle, but was stiff and saturated with blood from their wounds. A few days after their reception into the hospitals, tertian intermittent fever made its appearance, attacking many of the wounded, and, in the majority, retarding or completely arresting convalescence. On many of those severely wounded it exerted a decidedly pernicious influence, and no doubt contributed, in some cases, to a fatal termination. It not only attacked the wounded in the hospitals, but prevailed extensively in camp and among the population of the town and neighboring country. I cannot say to what extent, this may be attributed to the putrid exhalations arising from the numerous bodies of men and horses slain in the different combats, and which had been slightly covered with earth, and emitted a most sickening and offensive effluvia. This, doubtless, contributed largely towards infecting or destroying the purity of the air, and establishing a poisonous miasm.

With these preliminary remarks, I will now give you an outline of a few of the more interesting cases resulting from gun-shot wounds, received during the three days' attack on Monterey, and which came under my observation at the time. With a view to some order and classification, I will describe first those of the head and face.

*Case* 1.—Corporal Sherridan, 1st Infantry, was struck by a musket-ball on the anterior and central portion of the os frontis, destroying it for a distance of two inches. Considerable portions of the brain issued from the wound, and notwithstanding the severity of the case, the patient appeared to suffer little or none until the third or fourth day, when, coma supervening, followed by delirium, he died.

Numerous wounds of the scalp, accompanied in three cases by destruction of the periosteum and outer table of the skull, came under my observation, but presented nothing new or different in their character and progress from ordinary cases.

*Case* 2.—Private Redville, of the 3d Infantry, in passing a stone wall, received a wound in the right eye, as he supposed, from a fragment of stone broken from the wall by a cannon-ball, and which struck him with force sufficient to knock him down. I saw him two or three hours after the injury was received, and found his eyelids so much swollen, as to render it very difficult to ascertain the condition of the eye itself. In placing my finger over the inner canthus, I felt a sharp point, apparently of some hard substance. This I immediately extracted with a pair of common forceps, and found it to be a fragment of grape, three-quarters of an inch in length, and half an inch in width at the centre, of an oblong or elliptical shape. It was of copper, or an alloy of that metal, and had evidently been broken off by striking the wall. On examining the eyeball I found it uninjured, the fragment having passed between it and the inner canthus, and penetrated to the posterior wall of the orbit, destroying the lacrymal sac, the os unguis, and wing of the sphenoid bone. Considerable inflammation and suppuration followed, and although at the present time the wound has entirely healed, the pupil remains permanently dilated, and vision destroyed. This seems to indicate an injury of the optic nerve, which the missile from its length must have reached and destroyed.

*Case* 3.—Private Jones, of the same regiment, was wounded about the same time by a musket-ball striking him near the angle of the inferior maxilla, on the right side, fracturing the bone, passing directly through the tongue and the corresponding portion of the bone on the opposite side. The tongue was completely severed at its base, hanging only by a few muscular fibres. The patient was almost moribund when brought in, and died shortly from excessive hæmorrhage.

*Case* 4.—Major. L., commanding the 3d Infantry, received a wound from an escopette-ball directly in the centre of the upper lip. The ball passed obliquely backwards and to the left, tearing away the bony palate, aud completely destroying the upper maxilla and malar bone of that side, and, fracturing the condyle of the inferior maxilla, passed out behind the ear near the mastoid process. The velum pendulum palati was completely separated from its superior connections and rested on the tongue. The whole of the alveolar process, together with the teeth on the left side was carried away. To enable him to articulate, as well as swallow, I contrived to fasten up the pendulous palate by a stitch, and afterwards by a ligature placed around the remaining incisor tooth, with a view of afterwards endeavouring to effect a union with the parts from which it was torn. I subsequently secured it more completely by a strong ligature passed through it in two places, the ends being brought together, and by means of a probe carried up through the nostril and fastened with adhesive plaster to the forehead. Intense inflammation followed, involving the whole side of the head, and during several days pieces of bone were being constantly separated and discharged. The previous ill health of this officer rendered his case the more unpromising. He had suffered for two or three years from severe and repeated attacks of asthma, which had so enfeebled his general health that the least exposure or fatigue was attended by intense suffering and danger of death. Up to the present time nature has made but little recuperative effort, in consequence perhaps of an attack of intermittent fever, which, in many cases, thus acts in retarding the healing process.*

*Case* 5.—A private of Col. Hays' mounted Texan Rangers was wounded on the 21st in an attack made on the eastern side of the city. A copper grape shot striking him at the same point as in the pre-

* This officer died a few days afterwards.—M

ceding case, passed obliquely backwards and downwards, wounding the tongue and fracturing the lower jaw on the left side near its angle; then coursing along the neck, beneath the integuments and muscles, lodged near the insertion of the left sterno-cleido mastoid muscle into the clavicle, where it was cut out. Fragments of bone came away, and considerable inflammation, with difficulty of swallowing, followed, but the wound progressed favourably, and notwithstanding the size of the shot and destruction of parts, is at the present time nearly healed. His head is considerably drawn down, and a rigidity of the jaw, with inability to speak, remain.

*Case* 6.—The sergeant-major of the 5th infantry was wounded on the 22d, the ball entering near the same point as in the two former cases, but passing obliquely backwards and upwards *above* the roof of the mouth, and lodging near the articulation of the jaw on the right side, between the coronoid process and masseter muscle. It was subsequently extracted, and the wound at the present moment has entirely closed, leaving, however, as in the former case, more or less immobility of the jaw.

*Case* 7.—Private Lewis, of the 1st Mississippi Regiment, was wounded on the 22d September. The ball struck him at the lower point of the lobe of the ear, and posterior edge of the ramus of the inferior maxillary bone on the left side. After fracturing this bone midway between its angle and articulation, the ball passed transversely inwards, tearing away the back part of the palate, and came out through the right malar bone. This case progressed favourably, and the wound at the present time is nearly healed. Some deformity, arising from ossific matter thrown out in the union of the jaw, and a certain degree of immobility remain. The close vicinity of the carotid artery to the point of entrance of the ball, and its entire escape from injury, renders this case doubly interesting.

The next order of wounds are those of the neck, thorax and abdomen, many of which, of an interesting character, presented themselves during the engagements, but the limits of my letter warn me I must reserve them for a future occasion. I will, however, describe a few cases of wounds of the pelvis and bladder, presenting some singularity in the direction and force of the balls, and interesting in the nature and result of the injuries they inflicted.

*Case* 8.—Lieut. G———, 4th Infantry, was wounded in three places about the same time, on the morning of the 21st September. The most severe wound, however, was one, in which the ball, striking the upper and anterior portion of the thigh, entered the pelvis, wounded the fundus of the bladder, and passed out at the sacro-ischiatic notch. The femoral vessels, in the course of the ball, escaped being wounded in a most remarkable manner. The urine passing freely through the wound necessarily produced considerable infiltration and inflammation of the cellular tissue of the thigh. By changing his position so as to lie on the left side, and introducing a catheter, which was constantly maintained in the bladder, no more urine escaped through the wound, and the inflammation rapidly subsided. No unfavorable symptoms followed. The usual separation of the parts destroyed in the course of the ball took place, succeeded by a healthy suppuration, at the usual period of gun-shot wounds, and a hope was entertained by his friends of his speedy recovery. This hope was still more strengthened when, on the tenth day after the wound was received, the catheter, by some accident, became obstructed, and remained so some time before it was discovered, and on its withdrawal and re-insertion, upwards of *twelve ounces* of urine were drawn off, showing conclusively that the wound in the bladder must have entirely closed, to enable it thus to retain so large a quantity of fluid. The expression of his countenance, and cheerfulness of manner, would hardly have indicated any great pain or suffering. It was only on the twentieth day that any alarm was excited in the minds of his friends, by his suddenly being attacked by rigors, followed by fever and profuse night-sweats, which, notwithstanding all the means made use of, rapidly reduced his strength, and he expired on the night of the 13th October, and on the *twenty-second* day after being wounded. A post-mortem examination of this case would have proved highly interesting, showing how far wounds of this description, affecting internal hollow organs, may heal, and the manner in which a restoration of the parts destroyed takes place; but the pressure of professional duties at the time has prevented so desirable a finish to the history of the case.

*Case* 9.—Private Capers, of the Baltimore Battalion, was wounded early on the 21st September. The ball entered directly above the os pubis, and taking a direction downwards and obliquely backwards, wounding in its course the bladder, passed out of the pelvis between the sacrum and tuberosity of the ischium on the left side. It was found lodged between the integuments and glutei muscles, from which point I extracted it. Urine passed freely at the time, from the wound over the pubis, but ceased shortly after the introduction of the catheter, which was constantly maintained in the bladder, as in the former case. Very little tension or tenderness of the abdomen followed, nor any symptoms of peritoneal inflammation, showing that the ball had entered the bladder without wounding the peritoneum.* Neither were there any signs of extravasation or infiltration of urine, and but little or no febrile action. About the tenth day after its reception the wound over the pubis, which had by this time entirely closed, broke out again, discharging urine; this was shortly afterwards followed by the opening of that in the nates, made for the exit of the ball. Through the latter, both fæces and urine, passed, showing that sloughing had taken place, and a communication formed between the rectum and bladder. The contents of both of these were occasionally discharged from the anterior wound. The patient lin-

* The ball entered the bladder externally to the point were the peritoneum is reflected from the posterior wall of the abdomen upon the fundus of the bladder.—M.

gered in this miserable situation until the *sixteenth* day, when he expired, worn out by pain and suffering.

*Case* 10.—Private Young, of the 1st Tennessee Regiment, was wounded nearly at the same time and place as the above. The ball entered just above the os pubis, and about one inch to the right of the symphysis. It ranged diagonally across the pelvis, inclining downwards, wounding both the bladder and rectum, and passing out through the left sacro-ischiatic foramen, just above the os coccygis ; urine and fæces passed out from both orifices of the wound. When brought in, it was supposed, from his general appearance, that he would survive his wound but a very short time. A catheter was introduced immediately, which was retained with considerable difficulty. The wounds were dressed in the usual manner ; urine and fæces continued, however, to pass out of the wounds, attended by considerable irritation and febrile action. In this condition he lingered *twenty-three* days, when he expired, worn out, as in the case of Capers, by long-continued suffering.

Having given a brief description, of a few of the gun-shot wounds in the different assaults on Monterey, I will conclude my communication, with a statement of the number and results of the larger amputations, performed on those occasions. The total number in the three divisions of the army was *twenty-eight*, viz: *ten* in the first division, *four* in the second, and *fourteen* in the third or volunteer division. Twenty were performed on the field, or on the following morning, in the camp ; the remaining *eight*, at subsequent periods, varying from five to twenty days. *Twelve* of the number, including *two* in those taken prisoners and operated upon by the Mexican surgeons, proved fatal, and the remaining *sixteen*, have nearly or quite recovered. This average of mortality was not confined to our wounded. I was told by Dr. Hidalgo, surgeon in charge of the Mexican military hospital, that of *thirteen* amputations performed there, *five* had proved unsuccessful, and one case, that had recently been operated upon, appeared to me to be in a critical condition, but whether the patient died or recovered I have not learned.* In addition to unfavorable causes, not enumerated among those I have heretofore noticed, and from which the Mexicans were happily exempt, was the repeated removals to which our wounded were subjected. In carrying them from the field to the camp, a distance of three or four miles, they suffered greatly ; and the subsequent removal to town, still farther increased the pain and danger, and in one or two cases, evidently, was productive of a fatal termination.

With a few remarks, on the appearance and condition presented by the two cases of amputations of the thigh, performed by the Mexican surgeons, in their hospital alluded to above, I will close. One of these had been operated upon on the same day with the injury, and the other some four or five days after. Neither stump on examina-

* This case subsequently proved fatal.

tion, after the removal of the dressings, presented any unusual appearance; on the contrary, the flaps had been neatly adjusted and brought together, and kept so by a number of interrupted sutures and adhesive straps, encircling it in every direction, and adhesion had apparently taken place, in one case along the line of divided integuments. No one, judging by the external appearance of the wound, if we except a degree of paleness of the integuments of the flap and some fœtor, would have suspected the condition and extent of disease within. On dressing the first case and removing the lint and adhesive straps, which had become somewhat offensive, the edges of the flap receded or partially separated, so as to reveal a large cavity or excavation, the whole surface of which was dark and ill-conditioned, and from the centre projected the end of the bone. There were no signs or appearance of suppuration or granulation having ever taken place in the divided muscles; on the contrary, they appeared absorbed or attenuated by previous discharge, of which none existed at this time. The patient rapidly sunk, and died on the fourth day after his admission into the Division hospital.

Private Alexander, of the Baltimore Battalion, the other case, was brought to our hospital some two days after the one above. His stump presented nearly the same appearance as the first, with no indications whatever of the diseased condition within. Eleven days after his admission, the flap gave way, disclosing the same appearance as in the former case, with most intolerable fœtor. Gangrene rapidly extended, and he died on the *twelfth* day after his admission, and the *thirteenth* from the time of the operation.

Among other consequences arising from gun-shot wounds, in my hospital, were two cases of traumatic tetanus, both of which proved fatal. The first case manifested itself seven days after the injury, which was a wound of the knee-joint, with a fracture of the patella by a grape-shot. The man was brought from the camp of the 4th Infantry to the Division hospital, and was attacked a few hours afterwards, by opisthotonos, followed by trismus and severe spasmodic action of all the muscles of the body. He died the same night. The other case originated from a gun-shot wound of the left thigh, in which the ball passed down to the femur, six inches below the trochanters, and taking a direction upwards on the outer side of that bone, denuded it entirely of the periosteum for the distance of three or four inches, and was cut out from beneath the gluteus maximus muscle of the same side. Here the first semptoms manifesting an attack of this dreadful disease was violent spasmodic action of the muscles of the injured limb, which soon extended to those of the whole body, followed by trismus and a certain degree of opisthotonos. He expired on the *fifteenth* day after receiving his wound, and nine days after being received from the Mexican hospital; having been taken prisoner and carried thither on the 21st September, the day on which he was wounded,—*New York Jour. of Med.*

